# Polypropylene and Polylactic Acid Microplastics Alter Plateau Wetland Seed Bank Emergence and Community Assembly: A Greenhouse Stress Test Experiment

**DOI:** 10.3390/plants15060910

**Published:** 2026-03-15

**Authors:** Zhe-Xi Luan, Jia Ran, Hao-Qin Xiong, Hong Xiang, Xiao-Long Sun

**Affiliations:** 1 Yunnan Key Laboratory of Plateau Wetland Conservation, Restoration and Ecological Services, College of Ecology and Environment, Southwest Forestry University, Kunming 650224, China; 15203820860@163.com (Z.-X.L.); xionghaoqin@swfu.edu.cn (H.-Q.X.); 2 National Plateau Wetlands Research Center, Southwest Forestry University, Kunming 650224, China; 3 China Three Gorges Corporation Yunnan Branch, Kunming 650051, China; 15398520964@163.com

**Keywords:** microplastics, soil seed bank dynamics, plant diversity, plateau wetland, nitrogen dynamics

## Abstract

Microplastic contamination has emerged as a growing concern for soil ecosystems and their ecological functioning. However, the effects of microplastic contamination in fragile plateau wetlands remain poorly understood. In this study, biodegradable polylactic acid (PLA) and conventional polypropylene (PP) MPs were compared using soil seed bank collected from terrestrial and hygrophytic habitats in the Xingyun Lake plateau wetland. Seed germination, species diversity, and soil chemical properties were evaluated. Habitat specific effects were observed, and PLA induced stronger inhibition of seed germination and diversity than PP. These findings underscore the need to incorporate plant ecological responses, including germination physiology under chemical stress and soil seed bank dynamics, into ecological risk assessments of MPs. Furthermore, MP associated changes in nutrient dynamics and soil chemistry were examined, providing insight into potential long-term implications for plateau wetland restoration and plant community recovery.

## 1. Introduction

Widespread environmental pollution and ecological concerns have been driven by conventional plastic use worldwide. To mitigate these risks, biodegradable plastics (BPs) have been promoted as alternatives to conventional plastics [1]. BPs are polymeric materials designed to undergo microbially mediated degradation and to be mineralized into simple end products (e.g., CO_2_, H_2_O, and biomass) under defined conditions [2]. Accordingly, BPs are often regarded as environmentally friendly alternatives to conventional plastics. However, under natural conditions, biodegradable plastics may not fully mineralize [3]. Instead, fragmentation can occur during degradation, generating microplastics (MPs), which are commonly defined as plastic particles smaller than 5 mm [4,5,6]. MPs can accumulate in soils at concentrations exceeding 40,000 particles/kg [7]. MPs can also persist for extended periods [8], thereby posing potential risks to soil biota. Although BPs are designed to degrade more rapidly than conventional plastics and are expected to reduce long term environmental burdens, MPs generated during BP degradation may pose ecological risks comparable to, or greater than, those of conventional MPs in some settings [9,10].

The accumulation of MPs in natural ecosystems has become an increasing concern in plant ecology, particularly in plateau wetlands [11]. Wetlands occur at the interface between terrestrial and aquatic systems and can function as both sources and sinks of MPs through transport, deposition, and resuspension processes [12]. In wetland ecosystems, the soil seed bank is crucial for vegetation regeneration and ecosystem recovery, and seed bank dynamics are highly sensitive to changes in soil physicochemical properties [13,14]. A growing body of research has examined the toxicological impacts of MPs on plant growth [15]. However, the effects of MPs on soil seed bank dynamics in fragile plateau wetlands remain largely unexplored, particularly under contrasting habitat conditions. Understanding these processes is important for evaluating ecological risks of microplastics in wetland ecosystems. Here, the effects of MPs were examined, with a focus on seed bank dynamics, germination physiology under chemical stress, and implications for plateau wetland restoration.

## 2. Results

### 2.1. Effects of MPs on Soil Seed Bank Germination

**Figure 1:**
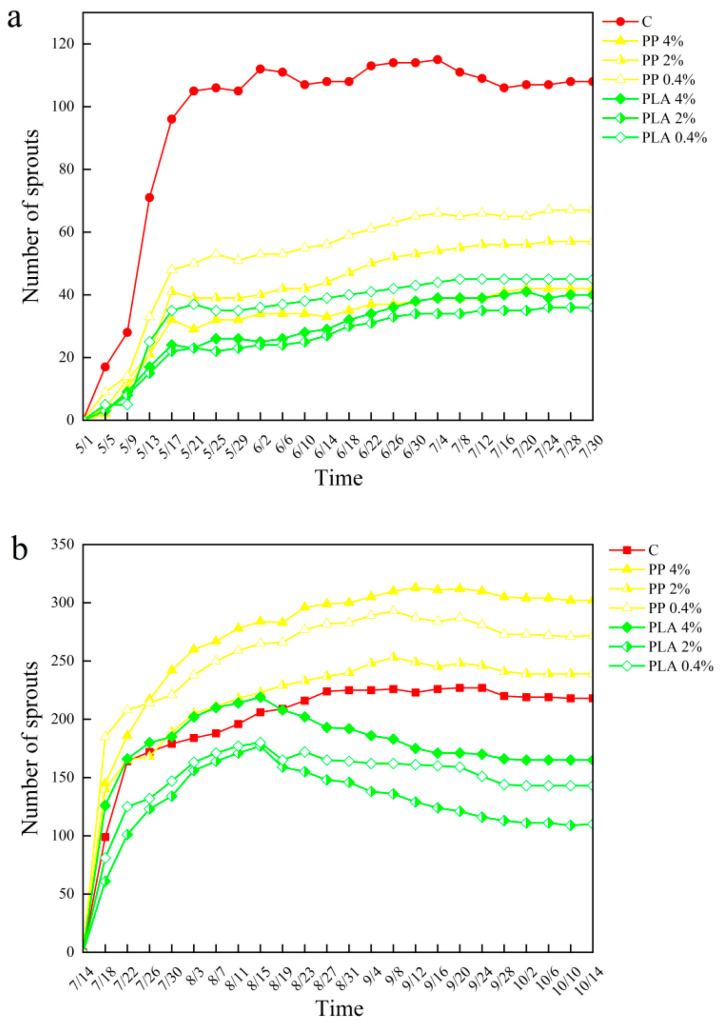
Effects of MP treatments on seed germination in terrestrial (**a**) and hygrophytic (**b**) soil seed banks. Lines show the cumulative number of germinated seeds recorded throughout the three month greenhouse experiment across MPs (PP and PLA) and concentrations (0.4%, 2%, and 4%), with C indicating the control. Control data were collected concurrently with the present experiment; related analyses have been reported previously [16].

In terrestrial soil, germination was markedly inhibited by PP MPs relative to the control (Figure 1). The number of sprouts decreased by 47–61%, with the strongest inhibition observed at 4% PP. PLA produced stronger suppression, with germination reduced by 58–67% across treatments. The reduction in germination increased with increasing PLA concentration. In hygrophytic soil, PP treatments enhanced seed germination compared with the control, with sprout numbers increasing by 10–39%, and the highest stimulation recorded at 4% PP. By contrast, germination was substantially suppressed by PLA at all concentrations, resulting in 24–45% reductions relative to the control. PLA tended to suppress germination more strongly than PP under the tested conditions. Germination responses differed by microplastic and habitat, and microplastic × habitat interaction was detected (Table 1). Table 1 summarizes germination outcomes, diversity indices, and soil chemical properties. These variables are discussed in the following subsections.

### 2.2. Effects of MPs on Species Composition in Soil Seed Bank

**Figure 2:**
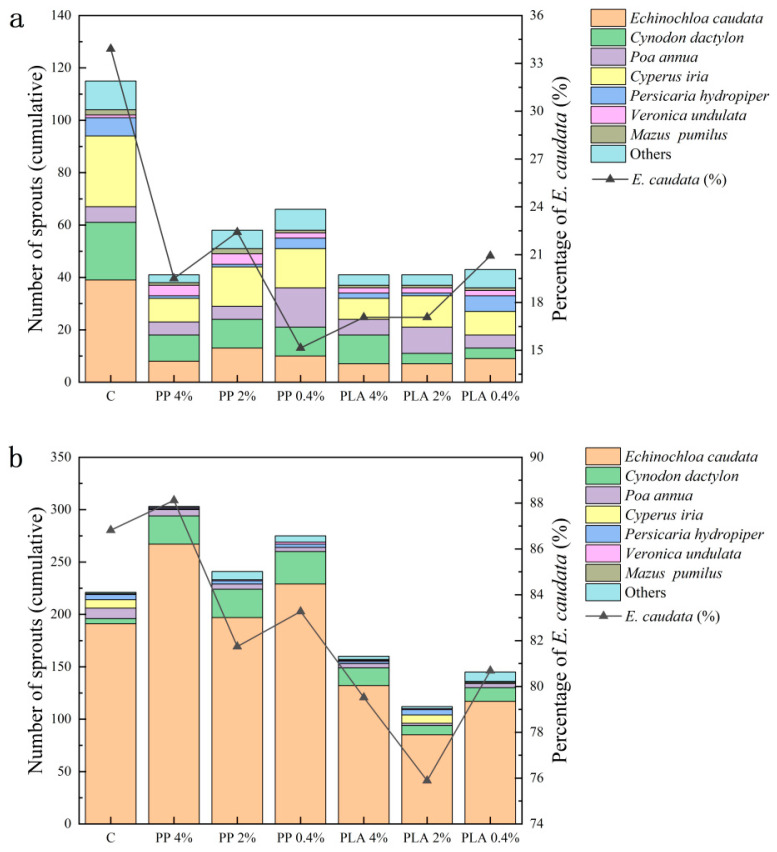
Community composition of common species and the percentage of the species dominant in the control (*Echinochloa caudata*) under PP and PLA treatments in (**a**) terrestrial and (**b**) hygrophytic soil seed banks. Stacked bars indicate the cumulative number of sprouts for common species on the left axis, and the black line denotes the percentage of *Echinochloa caudata* on the right axis.

MP exposure caused significant changes in seed bank community composition, with clear habitat dependent effects (Table 2 and Tables S1–S8). MP treatments were associated with shifts in dominance and changes in the contributions of less abundant taxa. Responses differed across habitats and MPs (Figure 2).

#### 2.2.1. Effects of MPs on Species Composition in Terrestrial Soil Seed Bank

In terrestrial soils, total emergence decreased relative to the control. The control produced 115 sprouts, and MP treatments reduced emergence by approximately 63–70%, yielding 41–66 sprouts (Tables S1 and S3). PLA treatments also reduced emergence by 63–65%, producing 41–43 sprouts. These shifts in total emergence were accompanied by changes in the densities of dominant species and the contributions of secondary taxa. These patterns indicate that both habitat and microplastic influenced community structure. In terrestrial soils, seed banks supported a broader range of taxa than hygrophytic soils. Under PP exposure, *Echinochloa caudata* was initially the most abundant species, but its dominance declined. For example, under PP 4%, *Cynodon dactylon* accounted for approximately 24% of total seedlings, and *Echinochloa caudata* contributed less than half of the control group’s density (Tables S1 and S2). Similarly, PLA treatments led to a decrease in *Echinochloa caudata* density. At PLA 4%, *Cynodon dactylon* became dominant and accounted for approximately 27% of total emergence (Tables S3 and S4).

MP treatments altered the relative abundance of dominant species. The control showed a high density of *Echinochloa caudata* (354.35 ± 100.45 seeds m^−2^). Under MP treatments, *Echinochloa caudata* density decreased. The lowest value observed at PP 4% (75.08 ± 10.40 seeds m^−2^) (Table S2). At the same time, *Cynodon dactylon* and *Cyperus iria* became more prominent. For example, at PP 4%, *Cynodon dactylon* accounted for approximately 24% of total seedlings (87.09 ± 20.81 seeds m^−2^). At PP 2%, *Cyperus iria* accounted for approximately 26% (132.13 ± 69.98 seeds m^−2^) (Figure 2). PLA treatments showed a similar trend, and *Echinochloa caudata* density decreased relative to the control (60.06 ± 13.76 seeds m^−2^ at PLA 2%). Meanwhile, *Cynodon dactylon* became the dominant species at PLA 4%, contributing approximately 27% to total emergence (99.10 ± 18.02 seeds m^−2^). Species richness in terrestrial seed banks remained relatively high, with 10–13 species in PP treatments and 11 species under PLA treatments.

#### 2.2.2. Effects of MPs on Species Composition in Hygrophytic Soil Seed Bank

In hygrophytic soils, emergence was higher than in terrestrial soils. The control yielded 220 sprouts. Under PP treatments, emergence increased by approximately 10–35% (Tables S5 and S7). In contrast, PLA treatments decreased total emergence by 50–55% relative to the control.

Hygrophytic seed banks showed strong dominance of *Echinochloa caudata*. Changes in community composition were driven more by total emergence rather than shifts in the dominant species (Figure 2). In the control, *Echinochloa caudata* accounted for approximately 76% to 81% of the total seedlings, with a density of 1697.78 ± 246.34 seeds m^−2^ (Tables S7 and S8). Under PP exposure, *Echinochloa caudata* remained dominant, accounting for approximately 82% to 88% of total seedlings. Meanwhile, PLA treatments reduced the density of *Echinochloa caudata*, with 749.63 ± 233.55 seeds m^−2^ at PLA 2% and 1170.37 ± 52.08 seeds m^−2^ at PLA 4% (Table S8). This decrease was accompanied by higher contributions from secondary species, including *Cynodon dactylon* and *Persicaria hydropiper*. These species did not replace *Echinochloa caudata* as the dominant species. In hygrophytic soils, treatment related changes were expressed mainly in dominant taxon density and secondary species contribution. Complete community turnover was not observed. PP treatments consistently produced higher total emergence than PLA treatments, with approximately 1.5–2.2 times more seedlings emerging under PP.

Taken together, MP exposure was associated with shifts in seed bank composition across habitats and MPs. Across both habitats, changes were most evident in relative contributions of dominant taxa. The compositional response was more pronounced under PLA than under PP.

### 2.3. Effects of MPs on Species Diversity in Soil Seed Bank

#### 2.3.1. Effects of MPs on Species Diversity in Terrestrial Soil Seed Bank

**Figure 3:**
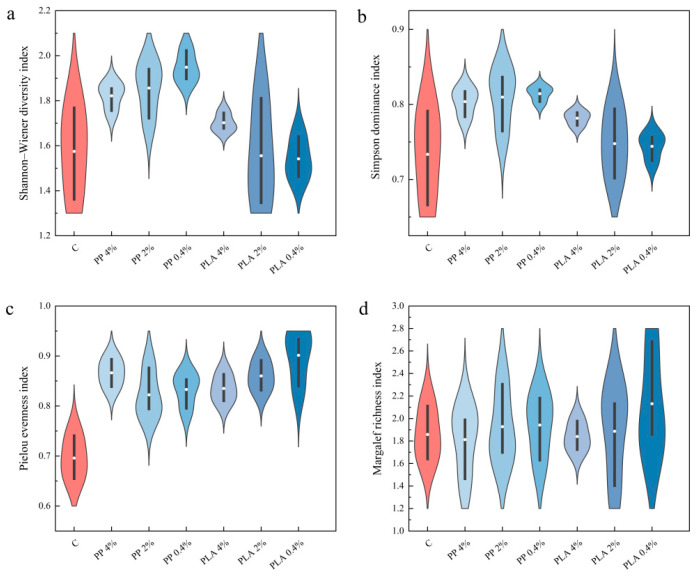
Effects of MP treatments on species diversity indices in terrestrial soil seed bank. (**a**) Shannon–Wiener diversity index; (**b**) Simpson diversity index; (**c**) Pielou evenness index; (**d**) Margalef richness index. Violin plots show the distribution of values across replicates (n = 3). The inner marker and bar indicate the central tendency and variability.

As shown in Figure 3, diversity responses in terrestrial seed banks differed across MPs and concentrations. At the same concentration, PP generally increased diversity, evenness, and richness more than PLA. Shannon–Wiener diversity index (H′) increased as PP concentration decreased and exceeded the control across PP treatments. Under PLA, H′ was lower than the control at 0.4% and 2%, with a small increase at 4%. Similarly, Simpson diversity index (D) increased under PP and PLA. The strongest increase occurred at low PP concentration. Pielou evenness index (E) increased under PP and PLA and tended to rise as concentration decreased. Margalef richness index (M) varied modestly, with a clearer increase at low concentration than at higher concentrations.

Two-way ANOVA supported these patterns. Habitat affected all diversity indices, and microplastic × habitat interactions were detected for H′ and D (Table 1). These results indicate that shifts in diversity and dominance differed between habitats. Pielou evenness index was influenced by microplastic and habitat. Margalef richness index was influenced mainly by habitat.

#### 2.3.2. Effects of MPs on Species Diversity in Hygrophytic Soil Seed Bank

**Figure 4:**
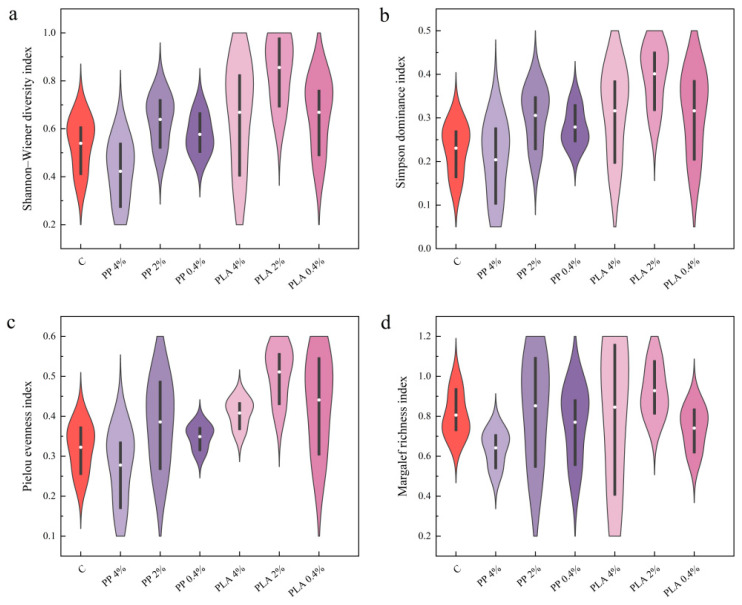
Effects of MP treatments on species diversity indices in hygrophytic soil seed bank. (**a**) Shannon–Wiener diversity index; (**b**) Simpson diversity index; (**c**) Pielou evenness index; (**d**) Margalef richness index. Violin plots show the distribution of values across replicates (n = 3). The inner marker and bar indicate the central tendency and variability.

As shown in Figure 4, hygrophytic seed bank exhibited lower diversity and greater sensitivity to microplastic treatments than terrestrial seed bank. In this habitat, diversity responses depended more on microplastic and concentration. Under PP, H′ decreased at the highest concentration, indicating a suppressive effect of high PP concentration. Lower PP concentrations produced smaller changes in H′. Under PLA, H′ was generally higher than the control and increased across concentrations in hygrophytic soils. Dominance patterns were consistent with H′. High PP concentration increased dominance of a few taxa, reflected by lower D. Medium and low PP concentrations, along with all PLA treatments, reduced dominance relative to the control. Contributions from non-dominant species increased. Pielou evenness index was lowest at high PP concentration but exceeded the control at lower PP concentrations. Under PLA, evenness was higher, with the clearest increase at the medium concentration. Richness responses were comparatively modest. Margalef richness index under PP was comparable to or lower than the control across concentrations. Under PLA, M peaked at the medium concentration, with weaker responses at both the high and low concentrations.

Habitat type affected all diversity indices, and microplastic × habitat interactions were observed for H′ and D (Table 1). These results indicate that changes in these indices differed between hygrophytic and terrestrial soils. Figure 3 and Figure 4 show these contrasts.

### 2.4. Effects of MPs on Soil Chemical Properties

**Figure 5:**
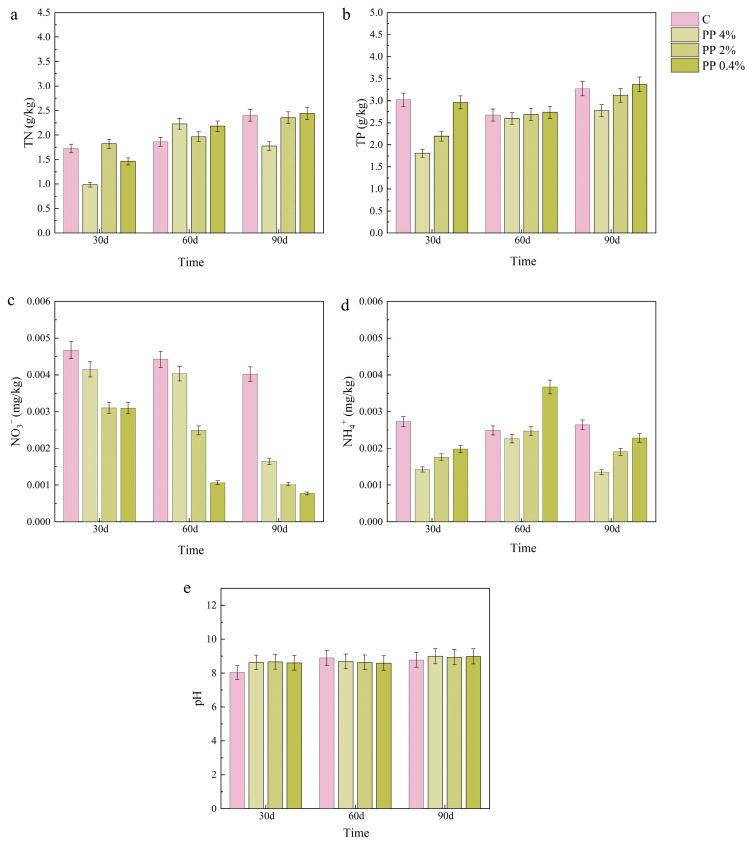
Effects of PP treatments on the chemical properties of terrestrial soils. Parameters include (**a**) total nitrogen (TN), (**b**) total phosphorus (TP), (**c**) nitrate nitrogen (NO_3_^−^-N), (**d**) ammonium nitrogen (NH_4_^+^-N), and (**e**) pH. Values are presented as mean ± SD (n = 3).

**Figure 6:**
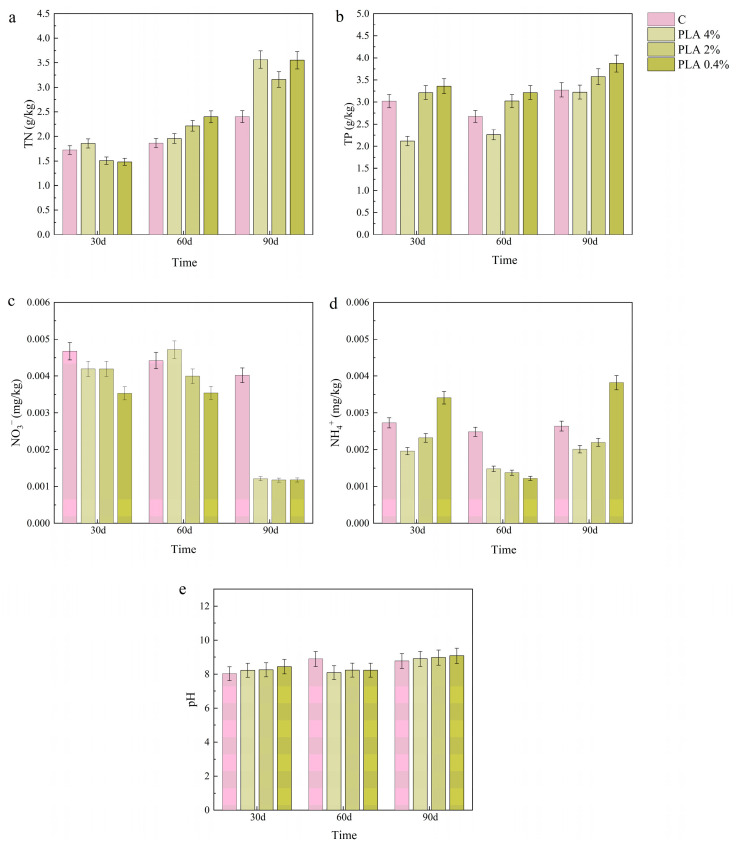
Effects of PLA treatments on the chemical properties of terrestrial soils. Parameters include (**a**) TN, (**b**) TP, (**c**) NO_3_^−^-N, (**d**) NH_4_^+^-N, and (**e**) pH. Values are presented as mean ± SD (n = 3).

**Figure 7:**
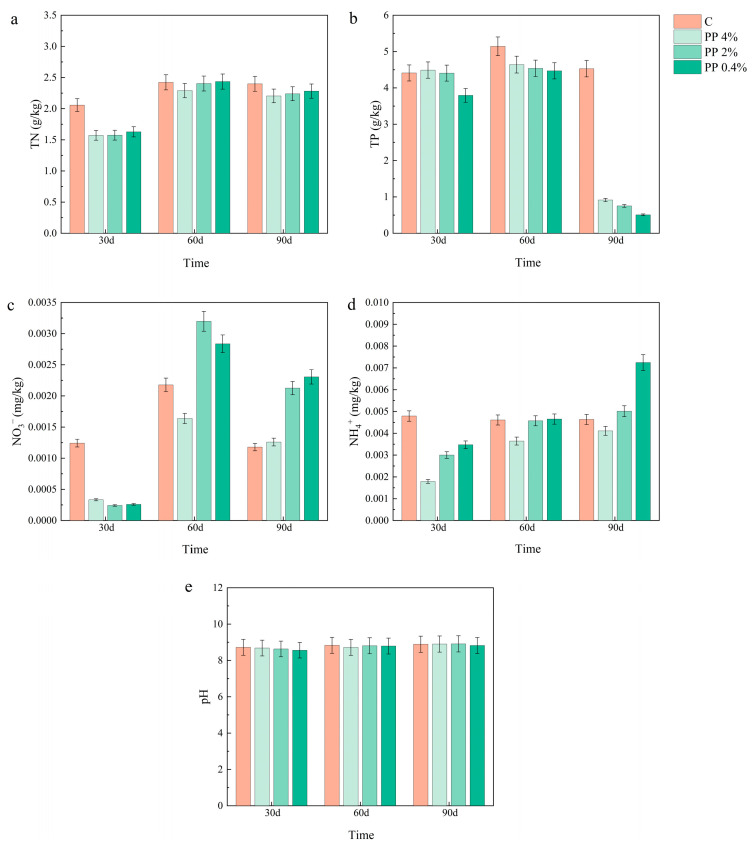
Effects of PP treatments on the chemical properties of hygrophytic soils. Parameters include (**a**) TN, (**b**) TP, (**c**) NO_3_^−^-N, (**d**) NH_4_^+^-N, and (**e**) pH. Values are presented as mean ± SD (n = 3).

**Figure 8:**
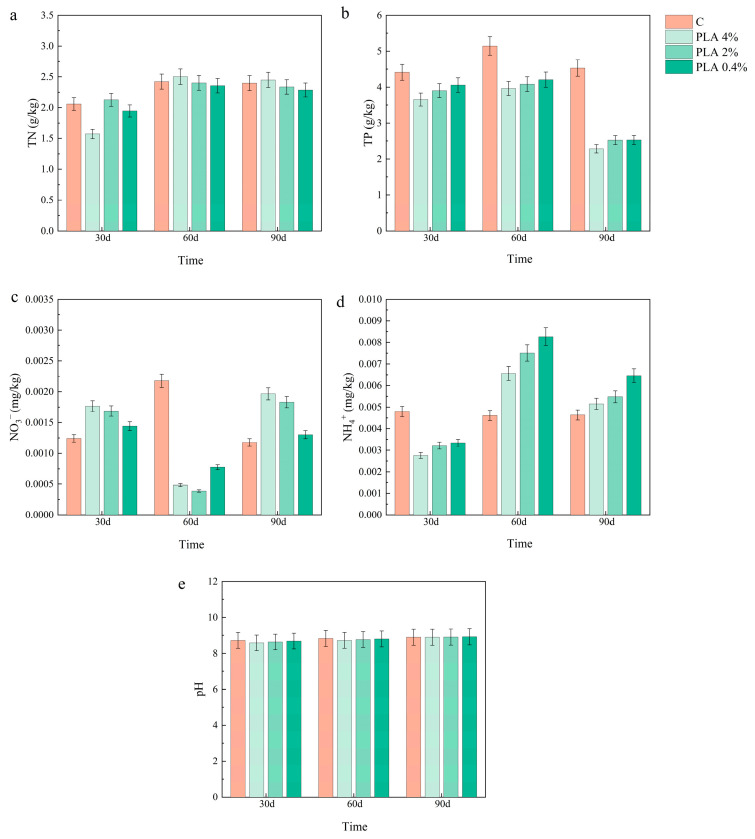
Effects of PLA treatments on the chemical properties of hygrophytic soils. Parameters include (**a**) TN, (**b**) TP, (**c**) NO_3_^−^-N, (**d**) NH_4_^+^-N, and (**e**) pH. Values are presented as mean ± SD (n = 3).

Soil chemical responses are shown in Figure 5, Figure 6, Figure 7 and Figure 8. At the end of the germination period (90 d), TN and TP were influenced by microplastic, habitat, and their interaction (Table 1). Ammonium nitrogen was influenced mainly by habitat, and nitrate nitrogen was influenced mainly by microplastic.

#### 2.4.1. Effects of MPs on Chemical Properties of Terrestrial Soil

The effects of MPs on the chemical properties of terrestrial soil are shown in Figure 5 and Figure 6. Total nitrogen in PLA-treated soils showed an upward pattern from 30 d to 90 d. The largest increase occurred under 4% PLA, and TN increased by approximately 25%. PP treatments induced only slight TN variation (less than 10%) over the same period. At 30 d, TN in the 4% PP group was approximately 20% lower than in other treatments. By 90 d, this difference was no longer evident. Total phosphorus decreased under PLA and PP. The decrease became larger as concentration increased. Under 4% PP and PLA, TP was 30–40% lower than the control at 30 d. Total phosphorus increased after 60 d but remained 10–15% lower than the control at 90 d. Under PP and PLA, NO_3_^−^-N decreased and remained 40–60% lower than the control across concentrations. The reduction was larger at 90 d. The effect was most evident in PLA-treated soils. For NH_4_^+^-N, levels increased with concentration in PLA treatments at 60 d. At the other sampling times, NH_4_^+^-N generally tended to decrease as MP concentration increased under both MPs. Moreover, NH_4_^+^-N levels in all MPs treatments were lower than in the control. At 30 d, levels were about 25% lower for PP and approximately 30% lower for PLA at 60 d. The impact on soil pH was most evident during the initial 30 d. PP treatments maintained pH values approximately 3–5% higher than PLA treatments. By 60–90 d, differences between treatments narrowed to less than 2%.

#### 2.4.2. Effects of MPs on Chemical Properties of Hygrophytic Soil

The effects of MPs on the chemical properties of hygrophytic soils are shown in Figure 7 and Figure 8. At 30 d, TN was approximately 15–20% lower than the control under 4% MP treatments for both PP and PLA groups. By 60 d, differences among treatments were small (generally below 5%), and by 90 d TN levels were close to the control. For TP, both PP and PLA treatments showed lower values than the control at 60 d and 90 d. Under 4% MPs addition, TP was approximately 25–30% lower than the control at 60 d and 35–40% lower at 90 d. This pattern was consistent with a sustained decline at longer exposure times and higher concentrations. In the PP treatment, soil NO_3_^−^-N decreased at 30 d, then increased, and decreased again. In the PLA treatment, the pattern was reversed: NO_3_^−^-N increased at 30 d, then decreased, and increased again. Despite different early-stage responses, both microplastic treatments showed higher NO_3_^−^-N levels than the control by 90 d. At 30 d, NH_4_^+^-N content was lower in all MP treatments, with reductions of 20–30% compared to the control. NH_4_^+^-N increased from 30 d to 60 d and 90 d in both PP and PLA soils. At 90 d, values were about 10–15% higher than at 60 d. Soil pH remained relatively stable across concentrations and sampling times, with variation within 2%.

### 2.5. Combined Effects of MPs and Habitat on Seed Germination, Soil Nutrients, and Community Diversity

The combined effects of microplastic and habitat on seed germination, soil nutrients, and community diversity are summarized in Table 1. Microplastic and habitat showed significant main effects and interactions (*p* < 0.05) for most variables, including number of sprouts, diversity indices, and key soil nutrients. PLA generally reduced germination and diversity indices compared with PP, and hygrophytic soils showed higher sprouting and nutrient contents than terrestrial soils.

**Figure 9:**
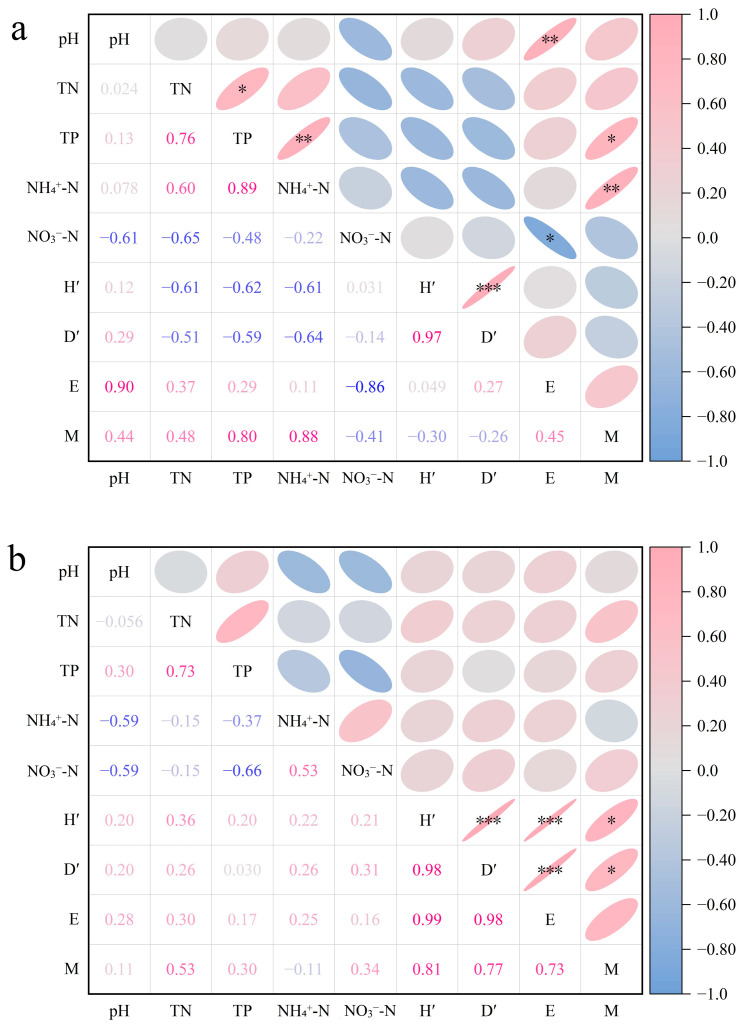
Pearson correlation matrix between soil chemical properties and plant community diversity indices in (**a**) terrestrial soil and (**b**) hygrophytic soil. Colors and ellipses indicate correlation direction and strength (see color bar). Numbers show r values. Asterisks denote significance (* *p* < 0.05, ** *p* < 0.01, *** *p* < 0.001).

To examine associations among variables, Pearson correlations were calculated for terrestrial and hygrophytic soils (Figure 9). Correlation analysis indicated that the relationships between soil chemical properties and community diversity varied with habitat and MPs. Diversity indices showed consistent pairwise associations within each habitat. These results indicate coherence among the four community metrics.

In terrestrial soils, diversity indices showed positive correlations with pH and negative correlations with TP (Figure 9a). Higher pH and lower TP coincided with greater species diversity. These patterns matched the measured data. PP treatments showed slightly higher pH and lower TP and exhibited higher diversity than PLA treatments. Total nitrogen showed weak and nonsignificant correlations with diversity. This indicates a limited association with community variation.

In hygrophytic soils, correlations between soil chemistry and diversity were generally weak (Figure 9b). Any chemistry and diversity linkage was less apparent under the conditions tested.

## 3. Discussion

### 3.1. Habitat Differences in Seed Bank Emergence Under PP and PLA

In terrestrial soils, PLA and PP inhibited germination, and inhibition increased as concentration increased, consistent with previous reports [17,18]. One possible explanation involves microplastic blockage of seed coat pores [19]. Reduced pore permeability can limit water uptake, gas exchange, and nutrient transport. These results indicate physical obstruction by MPs. Physical obstruction can reduce seed germination in drier conditions with limited water availability. By contrast, PP promoted germination under hygrophytic conditions. These contrasts may reflect differences in water availability and microplastic degradation behavior. Hygrophytic soils produced more seedlings and earlier emergence than terrestrial soils. Higher moisture likely contributed to faster germination of dormant seeds [20].

PP has often been reported to inhibit germination [21,22]. However, higher moisture in hygrophytic soils may mitigate the adverse physical effects of PP. Elevated soil moisture may reduce PP particle accumulation at seed soil interface [23]. Reduced particle accumulation can alleviate physical obstruction to water uptake. Reduced physical constraint under higher moisture may contribute to improved germination under PP across concentrations. Moreover, PP particles can adsorb onto soil aggregates or be incorporated into the soil structure [5]. These processes may reduce direct contact between PP and seeds. Reduced contact can create more favorable conditions for germination. Meanwhile, high humidity may accelerate PLA hydrolysis [24]. Hydrolysis may release water soluble intermediates and lead to local acidification. These changes may alter chemical conditions around seeds, reduce nutrient availability, and inhibit early seedling development [25,26,27]. These effects may increase germination suppression and seedling mortality under PLA compared with PP. This comparison reflects the combined effects of polymer chemistry and the physical form of the purchased particles. The SEM images of pristine particles indicate different morphologies between PP and PLA under the imaging conditions (Figure S3).

This study used natural soils to simulate realistic seed bank germination, but most environmental MPs are aged rather than pristine. Weathering may enhance additive leaching and alter particle surfaces [28]. Germination and early establishment responses to aged MPs may differ from responses to pristine MPs [29]. Therefore, future work should focus on the ecological effects of aged MPs on soil seed bank and plant communities under natural weathering conditions.

### 3.2. Dominant Species Shifts and Possible Consequences for Early Community Assembly

MP pollution reshaped seedling composition in plateau wetland seed banks. Annual herbs became more prevalent, and effects increased at higher concentrations, particularly in hygrophytic soils (Table 2). These findings are consistent with evidence that MPs can reshape plant community structure [30]. MPs can reduce stability and niche breadth of dominant species and can act as a selective filter during early community assembly. In this study, the selective filter was expressed as a shift toward annual herbs at higher MP concentrations. The increased dominance of species such as *Echinochloa caudata* and *Cyperus iria* may reflect their high germination plasticity and short life cycles. These traits can support rapid use of transient resources under unstable conditions [31]. Evans et al. reported similar patterns and suggested that annual dominated seed banks can be relatively resistant to environmental stress under fluctuating conditions [32]. Because MPs can impose both physical and chemical stresses on germination and early establishment [15,33]. Differences in stress tolerance may contribute to dominance shifts toward annual herbs under MP exposure. Life form shifts may weaken ecosystem self-regulation and stability [34]. These shifts may influence long-term restoration outcomes.

In hygrophytic soils, *Echinochloa caudata* remained dominant, but the species declined under PLA. This pattern suggests cumulative chemical stress under PLA. One possible source of stress is PLA hydrolysis [35]. Hydrolysis may release lactic acid oligomers and low molecular weight organic acids. These degradation products may contribute to soil local acidification, potentially contributing to inhibited root elongation and seed germination [36]. These effects may occur even in wetland adapted species. Our study suggests that PLA degradation may exacerbate stress in key wetland species. Further research is required to test this interpretation.

In summary, PLA degradability may not alleviate stress on dominant wetland species and may increase ecological risks. These results underscore the need for nuanced assessments of biodegradable plastics. Their effects on ecosystem stability, species regeneration, and vegetation succession may be more complex than anticipated [37]. Given these findings, future research should examine long-term ecological implications of biodegradable MPs. Key topics include wetland ecosystem recovery and species interactions under field aged conditions.

### 3.3. Diversity Patterns Under MPs Across Habitats

In ecological theory, disturbance can promote species diversity by increasing environmental heterogeneity and reducing competitive exclusion. This idea aligns with the intermediate disturbance hypothesis [38]. Under intermediate disturbance levels, more species can coexist. Competitive dominance can weaken, and niche opportunities can expand, leading to higher diversity [39]. Therefore, the increased diversity indices observed in the PP treated terrestrial soils may reflect disturbance associated with MPs. Disturbance can reduce dominance and create opportunities for subordinate species [30].

In hygrophytic soils, PLA increased H′ and E at all concentrations. These increases coincided with declines in total germination and seed density of the dominant *Echinochloa caudata*. These results suggest that higher diversity under PLA may reflect “apparent diversity”. This interpretation is supported by the rank abundance curves in hygrophytic soils (Figure S1), which show a slightly flatter abundance distribution under PLA treatments than under CK. Diversity indices can rise when dominance weakens under stress and recruitment declines. Higher indices may not indicate improved community function or broader establishment [40]. Accordingly, we hypothesize that PLA-related stress, potentially linked to degradation processes, may shift relative abundances and suppress dominant species [30]. In systems typically dominated by few species, abnormal rises in E may reduce functional stability [41]. The increased diversity observed in hygrophytic soils under PLA exposure likely reflects stress and disturbance rather than an improvement in community function. Hygrophytic seed bank diversity remained lower than terrestrial diversity. This pattern may reflect hydrological filtering. High moisture can limit dispersal, germination windows, and oxygen availability. Only flood tolerant species may persist under these conditions. Richness and heterogeneity can decrease, and habitat differentiation can strengthen [42].

Many studies have focused on individual plant growth and physiological responses under MPs. Effects on seed bank processes and community diversity remain less explored. This study extends current evidence by focusing on seed bank dynamics across habitats. The results provide new insight into microplastic effects on ecosystem processes.

### 3.4. Soil Physicochemical Properties and Their Effects on Seed Bank Dynamics

Baseline soil chemistry was not measured before treatment application. Therefore, soil chemistry is interpreted as between treatment differences relative to the concurrent control at each sampling time. Across both habitats, PLA was consistently associated with higher TN but lower NO_3_^−^-N (Figure 5 and Figure 6). This pattern may be related to the greater susceptibility of PLA to hydrolysis. During this process, PLA may release labile carbon, including lactic acid and low molecular weight oligomers. These compounds may stimulate microbial activity and alter nitrogen transformation pathways in soil and sediment systems [43,44]. PLA exposure was also accompanied by a decline in soil pH. Lower pH may reduce the conversion of NH_4_^+^-N to NO_3_^−^-N, and this change may partly explain the lower NO_3_^−^-N under PLA [45,46]. High moisture may further promote water penetration and ester bond cleavage, thus enhancing PLA hydrolysis. Similar patterns have been reported in subcritical water experiments and soil incubation studies [47,48]. Taken together, these processes may shift nitrogen away from NO_3_^−^-N and toward less available or microbially bound pools. This shift may be one factor contributing to the stronger germination inhibition under PLA because nitrate can promote seed germination [49]. However, degradation intermediates and nitrogen transformation rates were not quantified in this study. Therefore, these mechanisms should be regarded as plausible interpretations rather than direct evidence. Both MPs reduced TP in soils. Lower TP coincided with higher diversity in terrestrial soils (Section 4.3). These patterns indicate that phosphorus limitation associated with microplastics may indirectly regulate seed bank composition [50].

Studies have shown that soil microplastic pollution alters soil chemical properties and nutrient cycling, subsequently affecting plant nutrients [26,51]. In this experiment, soil chemistry and diversity were linked. PLA was associated with lower NO_3_^−^-N and TP in terrestrial soils, and PP was associated with higher pH. These patterns were qualitatively consistent with Section 4.3. Diversity tended to be higher under lower phosphorus and slightly higher pH [52,53]. TN showed limited explanatory power for diversity patterns (Figure 9). In hygrophytic soils, links between soil chemistry and diversity weakened or disappeared. Hydrological filtering may override chemical signals associated with MPs [54,55].

Although MPs did not trigger catastrophic short-term shifts in soil chemistry, except for the apparent PLA-associated perturbation of N cycling, soils may exhibit a certain buffering capacity that mitigates such effects [56]. From a broader risk perspective, potential accumulation of MPs in soils should not be overlooked, particularly for biodegradable PLA. These considerations highlight the need for long-term monitoring and further research. Future work should evaluate persistence or intensification of changes in key biogeochemical processes, including nitrogen cycling.

### 3.5. Limitations and Future Work

1. 1. Ecological risks of PLA and PP

Taken together, the results indicate that PLA may pose ecological risks comparable to or greater than those of conventional MPs under the tested conditions. However, this inference is intended as a site and material specific warning rather than a general conclusion applicable to all environments. In future work, broader ranges of microplastic composition and size should be evaluated across habitats and environmental conditions.

2. Mechanistic hypotheses

Mechanistic interpretations in this study, such as acidification and microbially mediated nitrogen transformations, are treated as hypotheses. Future research should focus on quantifying microbial biomass, community features, and N-process rates (e.g., nitrification and denitrification). These measurements will be needed to confirm the proposed mechanisms and to improve understanding of microplastic effects on nutrient cycling.

3. Dose dependence and spatial replication

For presentation purposes, concentrations were pooled in Table 1 to highlight the main contrasts between microplastics and habitats. Dose dependence should be tested using a broader range of concentrations, and spatial replication should be extended across multiple sites. This approach will clarify dose effect relationships and influence of concentration on seed bank dynamics and plant responses across habitats.

4. Site specific considerations

Because all plots were located within the same wetland, similarity in seed bank composition and abundance among plots is expected. Site specific processes such as hydrological transport, wind dispersal, and animal movement may contribute to spatial patterning over short distances. Our results should be interpreted primarily as site scale evidence of the observed trends. Future research would benefit from increasing the number of independent plots distributed across a broader geographic area and with greater inter plot spacing. Robustness and generalizability would be improved by this design.

5. Particle surface properties and additives

We interpret the PP versus PLA contrasts mainly in terms of polymer chemistry. Particle surface properties and additive composition were not measured in this study. These factors may influence microplastic effects on soils and seed bank responses. Future work should include surface characterization and additive screening to refine the attribution of observed effects. By examining these factors, we can better understand the contributions of both polymer chemistry and physical properties to the ecological impact of microplastics.

## 4. Materials and Methods

### 4.1. Soil Sample Collection

Soil samples were collected from the eastern shore of Xingyun Lake (E 102°48′1.14″, N 24°19′44.38″) on the Yunnan Plateau in Southwest China. This site represents a typical plateau wetland ecosystem, characterized by diverse vegetation and moderate human disturbance. Because plastic mulch film is used in vegetable cultivation around Xingyun Lake, plastics and MPs can be transported into the plateau wetland via surface runoff. Sampling was conducted in March 2023. Sites were classified as terrestrial or hygrophytic based on predefined thresholds of field moisture and distance to water (Table S9). Within each habitat (i.e., terrestrial or hygrophytic), three 10 m × 10 m plots were randomly established as spatial replicates, with a minimum spacing of 5 m between plots. Within each plot, five 4 m × 4 m subplots were randomly established. From each subplot, ten soil blocks (20 cm × 25 cm × 10 cm) were randomly collected from the surface soil (0–10 cm). For each plot, soils from the five subplots were pooled to form one composite soil. Thus, each habitat had three independent plot level composite soils. These plot level composites were treated independently and served as the statistical replicates. These composite soils served as the germination substrate for the corresponding treatments. All collected soils were air dried, manually cleared of visible roots, stones, and debris, and stored until further use.

### 4.2. Material Selection and Sample Processing

PLA and PP were selected as the representative microplastic types for the experiment. All MPs were obtained from a commercial supplier (Kaison Plastic Technology Co., Ltd., Deyang, China), with a nominal particle size of 150 μm. Fourier transform infrared spectroscopy (FTIR, Nicolet iS20, Thermo Fisher Scientific, Waltham, MA, USA) was used to confirm polymer identity (Figure S2). Scanning electron microscopy (SEM, S-4800, Hitachi High-Tech Corporation, Tokyo, Japan) was used to examine the morphology of the pristine particles (Figure S3). No obvious agglomeration was observed during sample preparation. Microplastic concentrations were selected to span a broad gradient from commonly used experimental loadings [57,58] to highly contaminated topsoil hotspot conditions reported to reach up to approximately 7% (*w*/*w*) [59]. MPs were added at 0.4%, 2%, and 4% (*w*/*w*; dry soil basis), hereafter referred to as low, medium, and high loadings. The two higher concentrations (2% and 4%) were used as stress test scenarios simulating upper bound or hotspot conditions, rather than representing typical environmental exposure. Although these concentrations are relatively high compared with field conditions, they were selected to evaluate potential effects under extreme contamination scenarios on soil ecosystems and seed bank dynamics. The selected particle size (150 μm) is environmentally relevant for soil systems, and particles of this size have been reported to exert negative effects [60,61]. Preliminary tests indicated low variance among replicates, supporting the robustness of the subsequent statistical analyses.

### 4.3. Seed Germination Experiment

For terrestrial soil, germination trays with drainage holes (37 cm × 30 cm × 7.5 cm) were used, whereas trays without drainage holes (37.5 cm × 30 cm × 10 cm) were used for hygrophytic soil. In all trays, a 5 cm thick nutrient substrate layer was placed at the bottom to minimize interference from non-seed bank species. Each 1 kg soil sample was thoroughly mixed with the designated microplastic type and concentration. The treated soil was then evenly spread over the basal substrate as an approximately 1 cm thick layer to simulate the natural surface accumulation of MPs in soils [62,63]. The trays were placed in a greenhouse under controlled conditions. All treatments were conducted concurrently in the same greenhouse under identical conditions. The no microplastic control group was run concurrently in the same greenhouse campaign and partially overlaps with a related publication [16]. This overlap is limited to the baseline control treatment, including the control group emergence data and the associated soil chemistry measurements collected in the same campaign. No microplastic treatment data were shared between the two studies. Temperature was maintained between 18 and 30 °C. Soil moisture was kept at 55–65% for terrestrial soil and 80–100% for hygrophytic soil, corresponding to field conditions in the respective habitat types around Xingyun Lake. Trays were randomly positioned and locations were rotated at each counting time to minimize potential within greenhouse spatial heterogeneity. Seed germination was monitored weekly, and newly emerged seedlings were counted and identified. Because a natural soil seed bank was used, germination was defined as the visible emergence of the radicle or shoot above the soil surface. Emerged species were identified using hypocotyl and cotyledon morphology, together with regional floristic references [64]. Identified seedlings were carefully removed to prevent competition with ungerminated seeds. Unidentified individuals were transplanted into separate trays for continued growth until taxonomic identification was possible. Seedling emergence was monitored for three months. An additional one-month observation period was conducted. No new seedlings emerged during this period, indicating that germination had ceased.

### 4.4. Measurements and Calculations of Seed Bank and Soil Chemical Properties

#### 4.4.1. Seed Bank Density and Germination Metrics

Seedling emergence data were used to estimate soil seed bank density (seeds m^−2^). Soil seed bank density was defined as the number of emerged seedlings divided by the surface area of the sampling unit. Germination was recorded over time, and statistical comparisons were based on final cumulative emergence.

#### 4.4.2. Alpha Diversity Indices

Alpha diversity indices were used to assess seed bank diversity, including:

$$\mathrm{Shannon}-\mathrm{Wiener}\;\mathrm{diversity}\;\mathrm{index}\;(H^{\prime }):\;H^{\prime }=-\mathrm{Pi}\;\mathrm{lnPi}$$

$$\mathrm{Simpson}\;\mathrm{diversity}\;\mathrm{index}\;(D):\;D=1\;-\sum {\mathrm{Pi}}^{2}$$

Margalef richness index (M): M=(S−1)/lnN

Pielou evenness index (E): E=H′lnS

Here, N is the total number of individuals, S is the number of species, and Pi is the proportion of individuals belonging to species i.

Diversity metrics were calculated from emerged seedlings and therefore represent the germinable fraction of the seed bank under the experimental conditions.

#### 4.4.3. Soil Chemical Properties

Soil samples were collected from each germination tray on days 30, 60, and 90 after experiment initiation to assess changes in chemical properties. Total nitrogen (TN) was determined using the Kjeldahl method [65], and total phosphorus (TP) was analyzed by the ammonium molybdate colorimetric method after acid digestion [66]. Nitrate nitrogen (NO_3_^−^-N) and ammonium nitrogen (NH_4_^+^-N) were extracted with potassium chloride (KCl, AR, Sinopharm Chemical Reagent Co., Ltd., Shanghai, China) and quantified using a continuous flow analyzer (AA3, SEAL Analytical, Norderstedt, Germany) [67]. Soil pH was measured using a glass electrode (Starter 3100, OHAUS Corporation, Parsippany, NJ, USA) in a 1:2.5 soil to water suspension. Baseline soil chemistry was not measured before treatment application. Results are interpreted as between treatment differences at each sampling time. Although baseline soil data were not available, plot-level replication and uniform experimental conditions minimized potential initial heterogeneity.

### 4.5. Data Analysis

For each habitat, there were three independent replicates (n = 3), and each replicate came from one independent plot. Treatment effects were assessed using two-way analysis of variance (ANOVA), with microplastic and habitat as fixed factors, followed by Tukey’s honestly significant difference (HSD) test (*p* < 0.05). Results are presented as the mean ± standard deviation (SD). Correlations between soil chemical properties and community indices were examined separately for terrestrial and hygrophytic soils using Pearson’s correlation. Statistical analyses were performed using IBM SPSS Statistics 27 (IBM Corp., Armonk, NY, USA). Figures were prepared using OriginPro 2025 (OriginLab Corporation, Northampton, MA, USA), and data were organized using Microsoft Excel (Microsoft Corp., Redmond, WA, USA).

## 5. Conclusions

This study suggests that PLA microplastics may exert stronger effects on the seed bank than PP. Effects were observed for seed germination, species diversity, and community composition. These findings indicate that biodegradable microplastics may pose ecological risks in wetlands. Ecosystem stability and function may be affected under high moisture conditions. PLA is often considered a safer alternative to conventional plastics. In this study, plant ecological processes may still be disrupted under PLA. Seed germination and community dynamics may be altered. Dominant species may be suppressed, and community resilience may shift.

Although this study does not directly measure microbial interactions or long-term ecological changes, the results provide a basis for the hypothesis that PLA may influence wetland plant health and species regeneration. Shifts in diversity observed in hygrophytic soils suggest that microplastic exposure may promote changes in plant community composition. And ecosystem functioning may be affected.

Future research should investigate long-term effects of biodegradable microplastics on wetland plant communities. Particular emphasis should be placed on functional traits, germination physiology under stress, and seed bank processes. Additionally, interactions between microplastics and soil microorganisms should be examined. Effects on nutrient cycling should be evaluated to improve understanding of consequences of microplastic pollution for wetland ecosystems.

## Figures and Tables

**Table 1:** The influence of microplastic and habitat on seed germination, plant community diversity, and soil chemical properties.

Measurement Index	Terrestrial		Hygrophytic		M	H	M × H
	PP	PLA	PP	PLA			
Number of sprouts	55.33 ± 12.58 c	40.33 ± 4.51 c	271 ± 31.51 a	139.33 ± 27.68 b	**	**	**
Shannon-Wiener diversity index	1.88 ± 0.07 a	1.60 ± 0.09 b	0.55 ± 0.11 d	0.73 ± 0.11 c	ns	**	**
Simpson diversity index	0.81 ± 0.01 a	0.76 ± 0.02 a	0.27 ± 0.05 c	0.35 ± 0.05 b	ns	**	*
Pielou evenness index	0.84 ± 0.02 a	0.87 ± 0.03 a	0.34 ± 0.05 c	0.45 ± 0.05 b	*	**	ns
Margalef richness index	1.88 ± 0.06 a	1.94 ± 0.16 a	0.76 ± 0.11 b	0.83 ± 0.09 b	ns	**	ns
TN (g/kg)	2.19 ± 0.36 c	3.43 ± 0.23 a	2.24 ± 0.04 c	2.35 ± 0.07 b	**	**	**
TP (g/kg)	3.09 ± 0.3 b	3.56 ± 0.32 a	0.73 ± 0.13 d	2.44 ± 0.15 c	**	**	**
NH_4_^+^ (mg/kg)	0.002 ± 0.0005 b	0.003 ± 0.001 b	0.006 ± 0.0013 a	0.006 ± 0.0006 a	ns	**	ns
NO_3_^−^ (mg/kg)	0.002 ± 0.0007 a	0.001 ± 0.0002 b	0.002 ± 0.0005 ab	0.002 ± 0.0002 ab	*	ns	ns
pH	8.99 ± 0.08	8.99 ± 0.09	8.88 ± 0.05	8.9 ± 0.02	ns	ns	ns

Values are mean ± SD (n = 3 plot level replicates). Different lowercase letters within the same row indicate significant differences among microplastic × habitat combinations (Tukey’s HSD test, *p* < 0.05). For each microplastic × habitat combination, the reported values are the mean across the three concentrations (0.4%, 2%, and 4%), emphasizing contrasts between microplastic and habitat. Detailed concentration-specific results are presented in several subsections of Section 2. M, main effect of microplastic; H, main effect of habitat; M × H, interaction. ns, not significant; * *p* < 0.05; ** *p* < 0.01.

**Table 2:** Comparison of dominant species percentage, life form, and density in terrestrial and hygrophytic soil seed bank under different MP treatments.

Treatment	Habitat Type	Dominant Species	Relative Abundance (%)	Life Form	Density (Seeds m^−2^)
C	Terrestrial	*Echinochloa caudata*	33.91	Annual herb	354.35 ± 10.45
	Hygrophytic	*Echinochloa caudata*	86.82	Annual herb	1697.78 ± 246.34
PP 4%	Terrestrial	*Cynodon dactylon*	24.39	Perennial herb	87.09 ± 20.81
	Hygrophytic	*Echinochloa caudata*	88.12	Annual herb	2370.37 ± 307.19
PP 2%	Terrestrial	*Cyperus iria*	25.86	Annual herb	132.13 ± 69.98
	Hygrophytic	*Echinochloa caudata*	81.74	Annual herb	1751.11 ± 111.38
PP 0.4%	Terrestrial	*Cyperus iria*	27.73	Annual herb	138.14 ± 63.28
	Hygrophytic	*Echinochloa caudata*	83.27	Annual herb	2032.59 ± 312.17
PLA 4%	Terrestrial	*Cynodon dactylon*	26.83	Perennial herb	99.10 ± 18.02
	Hygrophytic	*Echinochloa caudata*	79.52	Annual herb	1170.37 ± 52.08
PLA 2%	Terrestrial	*Cyperus iria*	29.27	Annual herb	69.07 ± 10.40
	Hygrophytic	*Echinochloa caudata*	75.89	Annual herb	749.63 ± 233.55
PLA 0.4%	Terrestrial	*Cyperus iria*	20.93	Annual herb	84.08 ± 5.20
	Hygrophytic	*Echinochloa caudata*	80.69	Annual herb	1034.07 ± 207.00

Values are mean ± SD (n = 3 plot level replicates). Relative abundance (%) refers to the proportion of individuals of the dominant species among all germinated individuals within each treatment.

## Data Availability

The datasets used and/or analyzed during the current study are available from the corresponding authors on reasonable request.

## Supplementary Materials

The following supporting information can be downloaded at: https://www.mdpi.com/article/10.3390/plants15060910/s1, Table S1: Species composition of the terrestrial soil seed bank under PP treatments. Table S2. Terrestrial soil seed bank density under PP treatments. Table S3. Species composition of the terrestrial soil seed bank under PLA treatments. Table S4. Terrestrial soil seed bank density under PLA treatments. Table S5. Species composition of the hygrophytic soil seed bank under PP treatment. Table S6. Hygrophytic soil seed bank density under PP treatments. Table S7. Species composition of the hygrophytic soil seed bank under PLA treatment. Table S8. Hygrophytic soil seed bank density under PLA treatments. Table S9. Baseline descriptors used to distinguish terrestrial and hygrophytic habitats at Xingyun Lake. Figure S1. Rank abundance curves of hygrophytic soil seed bank under CK and PLA treatments. Figure S2. FTIR spectra of pristine PLA (yellow) and PP (green) microplastic particles. Figure S3. SEM images of pristine PP (a) and PLA (b) microplastics. Images were acquired at 6.00k (scale bar = 5 μm).

## Abbreviations

The following abbreviations are used in this manuscript:

MPs	Microplastics
BPs	Biodegradable plastics
PLA	Polylactic acid
PP	Polypropylene
TN	Total nitrogen
TP	Total phosphorus
NO_3_^−^-N	Nitrate nitrogen
NH_4_^+^-N	Ammonium nitrogen
H′	Shannon-Wiener diversity index
D	Simpson diversity index
M	Margalef richness index
E	Pielou evenness index
