# Supplementary material for: Polypropylene and Polylactic Acid Microplastics Alter Plateau Wetland Seed Bank Emergence and Community Assembly: A Greenhouse Stress Test Experiment
Source: Plants (Basel). 2026 Mar 15;15(6):910. doi: 10.3390/plants15060910 (PMC13029698; doi:10.3390/plants15060910)
Supplement: Supplementary file 1 [file plants-15-00910-s001.zip › plants-4128730-supplementary.pdf]

Table S1. Species composition of the terrestrial soil seed bank under PP treatments.

Species name	Family	Plant life form	Number of sprouts			
			C	PP 4%	PP 2%	PP 0.4%
Echinochloa caudata	Poaceae	A	39	8	13	10
Cynodon dactylon	Poaceae	P	22	10	11	11
Poa annua	Poaceae	A	6	5	5	15
Cyperus iria	Cyperaceae	A	27	9	15	15
Cyperus difformis	Cyperaceae	A	1	1	2	1
Pycreus flavidus var. *strictus*	Cyperaceae	A	0	1	0	1
Cyperus glomeratus	Cyperaceae	A	0	1	0	3
Persicaria hydropiper	Polygonaceae	A	7	1	1	4
Veronica undulata	Plantaginaceae	P	1	4	4	2
Mazus pumilus	Mazaceae	A	2	1	2	1
Epilobium hirsutum	Onagraceae	P	1	0	0	0
Ranunculus sceleratus	Ranunculaceae	A	1	0	0	0
Malva verticillata	Malvaceae	AB	1	0	0	0
Trigonotis peduncularis	Boraginaceae	B	1	0	0	0
Erigeron canadensis	Asteraceae	A	1	0	0	1
Rumex acetosa	Polygonaceae	P	1	0	0	0
Nasturtium officinale	Brassicaceae	P	1	0	0	0
Plantago asiatica	Plantaginaceae	P	1	0	0	1
Persicaria maculosa	Polygonaceae	A	1	0	1	0
Sida acuta	Malvaceae	ESS	1	0	0	0
Solanum nigrum	Solanaceae	A	0	0	1	0
Blumea balsamifera	Asteraceae	P/SS	0	0	1	0
Coreopsis grandiflora	Asteraceae	P	0	0	0	1
Coix lacryma-jobi	Poaceae	A	0	0	1	0
Laggera crispata	Asteraceae	P	0	0	1	0

P: Perennial herbaceous plant; B: Biennial herbaceous plant; A: Annual herbaceous plant; AB: Annual or biennial plant; SS: Subshrub; ESS: Erect subshrub; P/SS: Perennial herbaceous plant or Subshrub.

Table S2. Terrestrial soil seed bank density under PP treatments.

Species name \ Average density (seeds/m²)	C	PP 4%	PP 2%	PP 0.4%
Echinochloa caudata	354.35±100.45	75.08±10.40	114.11±27.52	93.09±31.64
Cynodon dactylon	195.20±130.03	87.09±20.81	102.10±34.11	99.10±23.84
Poa annua	51.05±46.23	45.05±23.84	45.05±32.48	135.14±32.48
Cyperus iria	240.24±65.17	78.08±72.26	132.13±69.98	138.14±63.28
Cyperus difformis	6.01±10.40	9.01±9.01	21.02±5.20	3.00±5.20
Pycreus flavidus var. strictus	-	6.01±5.20	-	3.00±5.20
Cyperus glomeratus	-	9.01±15.60	-	24.02±20.81
Persicaria hydropiper	60.06±52.01	6.01±10.40	6.01±10.40	36.04±32.48
Veronica undulata	9.01±9.01	36.04±9.01	36.04±32.48	18.02±9.01
Mazus pumilus	21.02±10.40	9.01±9.01	18.02±15.60	12.01±5.20
Epilobium hirsutum	3.00±5.20	-	-	-
Ranunculus sceleratus	3.00±5.20	-	-	-
Malva verticillata	9.01±15.60	-	-	-
Trigonotis peduncularis	3.00±5.20	-	-	-
Erigeron canadensis	3.00±5.20	-	-	3.00±5.20
Rumex acetosa	3.00±5.20	-	-	-
Nasturtium officinale	3.00±5.20	-	-	-
Plantago asiatica	3.00±5.20	-	-	3.00±5.20
Persicaria maculosa	3.00±5.20	-	3.00±5.20	-
Sida acuta	3.00±5.20	-	-	-
Solanum nigrum	-	-	3.00±5.20	-
Blumea balsamifera	-	-	-	3.00±5.20
Coreopsis grandiflora	-	-	3.00±5.20	-
Coix lacryma-jobi	-	-	3.00±5.20	-
Laggera crispata	-	-	3.00±5.20	-

Table S3. Species composition of the terrestrial soil seed bank under PLA treatments.

Species name	Family	Plant life form	Number of sprouts			
			C	PLA 4%	PLA 2%	PLA 0.4%
Echinochloa caudata	Poaceae	A	39	7	7	9
Cynodon dactylon	Poaceae	P	22	11	4	4
Poa annua	Poaceae	A	6	6	10	5
Cyperus iria	Cyperaceae	A	27	8	12	9
Cyperus difformis	Cyperaceae	A	1	0	0	3
Mazus pumilus	Mazaceae	A	2	1	1	1
Persicaria hydropiper	Polygonaceae	A	7	2	1	6
Veronica undulata	Plantaginaceae	P	1	2	2	2
Cyperus glomeratus	Cyperaceae	A	0	1	0	0
Pycreus flavidus var. *strictus*	Cyperaceae	A	0	1	0	0
Epilobium hirsutum	Onagraceae	P	1	1	0	0
Ranunculus sceleratus	Ranunculaceae	A	1	0	1	2
Malva verticillata	Malvaceae	AB	1	0	0	0
Trigonotis peduncularis	Boraginaceae	B	1	0	0	0
Erigeron canadensis	Asteraceae	A	1	0	0	1
Rumex acetosa	Polygonaceae	P	1	0	0	0
Nasturtium officinale	Brassicaceae	P	1	0	0	0
Plantago asiatica	Plantaginaceae	P	1	0	0	0
Persicaria maculosa	Polygonaceae	A	1	0	0	0
Sida acuta	Malvaceae	ESS	1	0	1	0
Solanum nigrum	Solanaceae	A	0	0	1	0
Eclipta prostrata	Asteraceae	A	0	0	1	0
Digitaria sanguinalis	Poaceae	A	0	1	0	0
Verbena officinalis	Verbenaceae	P	0	0	0	1

Table S4. Terrestrial soil seed bank density under PLA treatments.

Species name \ Average density (seeds/m²)	C	PLA 4%	PLA 2%	PLA 0.4%
Echinochloa caudata	354.35±100.45	66.07±36.41	60.06±13.76	78.08±28.96
Cynodon dactylon	195.20±130.03	99.10±18.02	36.04±23.84	39.04±13.76
Poa annua	51.05±46.23	54.05±32.48	93.09±34.11	45.05±9.01
Cyperus iria	240.24±65.17	75.08±22.67	69.07±10.40	84.08±5.20
Cyperus difformis	6.01±10.40	-	-	30.03±22.67
Pycreus flavidus var. strictus	-	3.00±5.20	-	-
Cyperus glomeratus	-	3.00±5.20	-	-
Persicaria hydropiper	60.06±52.01	9.01±9.01	12.01±5.20	54.05±39.27
Veronica undulata	9.01±9.01	21.02±13.76	21.02±22.67	21.02±13.76
Mazus pumilus	21.02±10.40	6.01±5.20	9.01±9.01	9.01±15.60
Epilobium hirsutum	3.00±5.20	3.00±5.20	-	-
Ranunculus sceleratus	9.01±15.60	-	3.00±5.20	21.02±20.81
Malva verticillata	3.00±5.20	-	-	-
Trigonotis peduncularis	3.00±5.20	-	-	-
Erigeron canadensis	3.00±5.20	-	-	3.00±5.20
Rumex acetosa	3.00±5.20	-	-	-
Nasturtium officinale	3.00±5.20	-	-	-
Plantago asiatica	3.00±5.20	-	-	-
Persicaria maculosa	3.00±5.20	-	-	-
Sida acuta	3.00±5.20	-	3.00±5.20	-
Solanum nigrum	-	3.00±5.20	-	-
Eclipta prostrata	-	-	6.01±10.40	-
Digitaria sanguinalis	-	-	3.00±5.20	-
Verbena officinalis	-	3.00±5.20	-	-

Table S5. Species composition of the hygrophytic soil seed bank under PP treatment.

Species name	Family	Plant life form	Number of sprouts			
			C	PP 4%	PP 2%	PP 0.4%
Persicaria hydropiper	Polygonaceae	A	5	1	3	3
Veronica undulata	Plantaginaceae	P	0	1	1	2
Echinochloa caudata	Poaceae	A	191	267	197	229
Cyperus iria	Cyperaceae	A	8	0	0	0
Cyperus microiria	Cyperaceae	A	0	1	7	5
Cynodon dactylon	Poaceae	P	5	27	27	31
Poa annua	Poaceae	A	10	6	5	4
Mazus pumilus	Mazaceae	A	1	0	0	0
Ranunculus sceleratus	Ranunculaceae	A	0	0	1	0
Plantago asiatica	Plantaginaceae	P	0	0	0	1

Table S6. Hygrophytic soil seed bank density under PP treatments.

Species name \ Average density (seeds/m²)	C	PP 4%	PP 2%	PP 0.4%
Persicaria hydropiper	38.52±18.50	8.89±8.89	23.70±5.13	23.70±22.37
Veronica undulata	-	5.93±5.13	2.96±5.13	11.85±10.26
Echinochloa caudata	1697.78±246.34	2370.37±307.19	1751.11±111.38	2032.59±312.17
Cyperus iria	68.15±18.50	-	-	-
Cyperus microiria	-	8.89±15.40	62.22±62.22	41.48±44.74
Cynodon dactylon	41.48±25.66	240.00±174.87	240.00±58.29	275.56±72.76
Poa annua	88.89±44.44	53.33±32.05	38.52±45.61	32.59±18.50
Mazus pumilus	2.96±5.13	-	-	-
Ranunculus sceleratus	-	-	8.89±8.89	-
Plantago asiatica	-	-	-	2.96±5.13

Table S7. Species composition of the hygrophytic soil seed bank under PLA treatment.

Species name	Family	Plant life form	Number of sprouts			
			C	PLA 4%	PLA 2%	PLA 0.4%
Veronica undulata	Plantaginaceae	P	0	1	1	0
Persicaria hydropiper	Polygonaceae	A	5	2	5	1
Echinochloa caudata	Poaceae	A	191	132	85	117
Cyperus niveus	Cyperaceae	A	0	1	0	4
Cyperus iria	Cyperaceae	A	8	8	8	1
Cyperus microiria	Cyperaceae	A	0	0	2	5
Cynodon dactylon	Poaceae	P	5	17	9	13
Poa annua	Poaceae	A	10	3	2	4
Mazus pumilus	Mazaceae	A	1	1	0	0
Ranunculus sceleratus	Ranunculaceae	A	0	1	0	0

Table S8. Hygrophytic soil seed bank density under PLA treatments.

Species name \ Average density (seeds/m²)	C	PLA 4%	PLA 2%	PLA 0.4%
Veronica undulata	38.52±18.50	14.81±18.50	41.48±27.16	8.89±15.40
Persicaria hydropiper	-	2.96±5.13	2.96±5.13	-
Echinochloa caudata	1697.78±246.34	1170.37±52.08	749.63±233.55	1034.07±207.00
Cyperus niveus	-	5.93±10.26	-	35.56±61.58
Cyperus iria	68.15±18.50	71.11±30.79	68.15±31.22	5.93±10.26
Cyperus microiria	-	-	17.78±30.79	38.52±28.57
Cynodon dactylon	41.48±25.66	151.11±46.19	80.00±8.89	115.56±72.76
Poa annua	88.89±44.44	20.74±35.92	14.81±13.58	35.56±32.05
Mazus pumilus	2.96±5.13	2.96±5.13	-	-
Ranunculus sceleratus	-	2.96±5.13	-	-

Table S9. Baseline descriptors used to distinguish terrestrial and hygrophytic habitats at Xingyun Lake.

Habitat zone	Elevation (m a.s.l.)	Soil moisture (%)	Distance to water (m, approximate)
Terrestrial	1724	55–65	100
Hygrophytic	1723	80–100	10

Field soil moisture denotes the typical moisture regime of each habitat and was used to set incubation moisture levels. Distance to water was quantified as the horizontal distance from the plot centroid to the nearest shoreline (waterline) using the measurement tool in Aowei Map based on the Jilin-1 remote-sensing imagery layer (data provider: Chang Guang Satellite Technology Co., Ltd.). Distances are reported as approximate values due to potential shoreline shifts associated with water-level variability and imagery acquisition time.

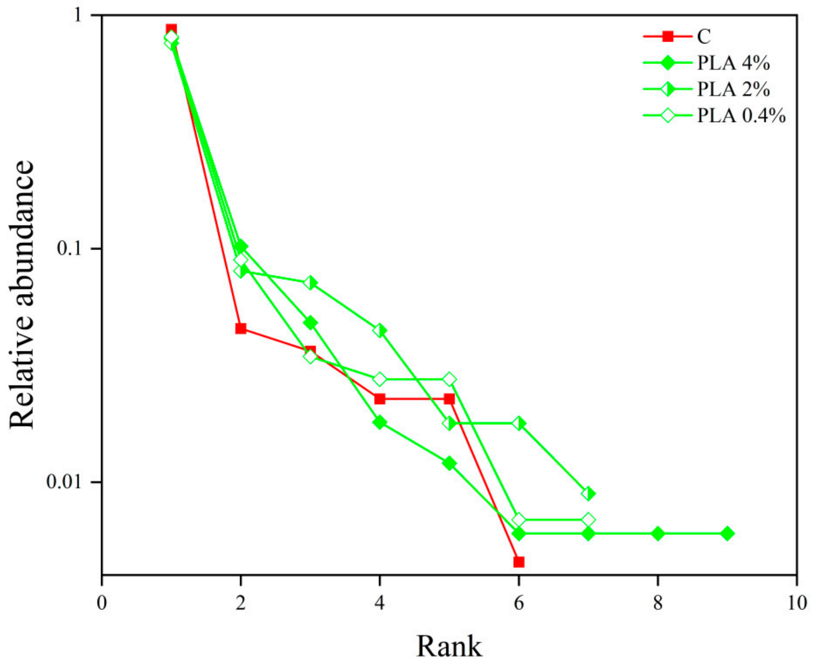

Figure S1. Rank abundance curves of hygrophytic soil seed bank under CK and PLA treatments. Species were ranked in descending relative abundance based on emergence counts. The y axis shows relative abundance on a log10 scale.

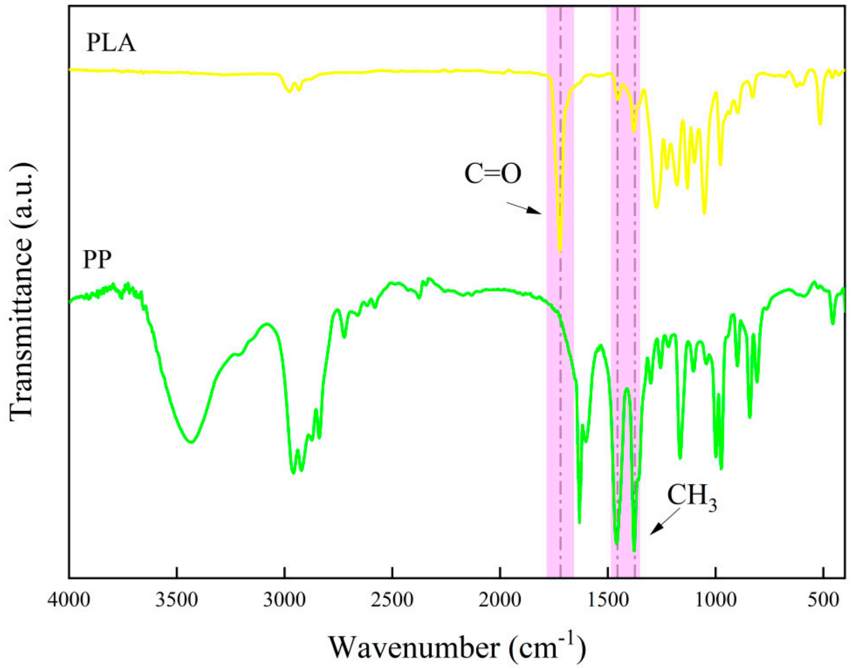

Figure S2. FTIR spectra of pristine PLA (yellow) and PP (green) microplastic particles. PLA shows a characteristic carbonyl (C=O) band at 1722 cm^{-1}. PP shows characteristic CH_3 bands at 1455 cm^{-1} and 1375 cm^{-1}.

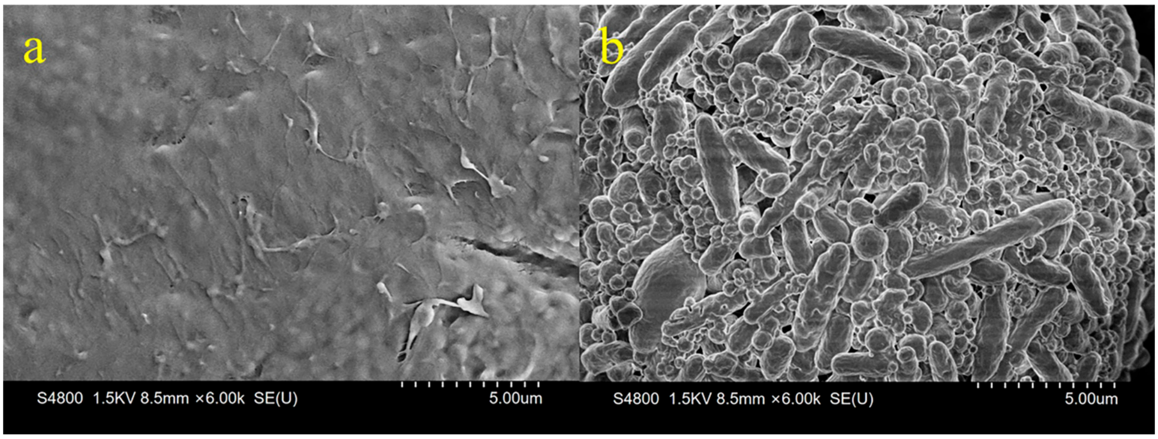

Figure S3. SEM images of pristine PP (a) and PLA (b) microplastics. Images were acquired at 6.00k (scale bar = 5 μm).
